# The challenges of estimating biological age

**DOI:** 10.7554/eLife.54969

**Published:** 2020-02-11

**Authors:** Alexey Moskalev

**Affiliations:** 1 Institute of Biology of the Komi Science CenterUral Branch of Russian Academy of SciencesSyktyvkarRussian Federation; 2 Syktyvkar State UniversitySyktyvkarRussian Federation; 3 Engelhard Institute for Molecular BiologyRussian Academy of SciencesMoscowRussian Federation

**Keywords:** biological age, longitudinal trajectory, mortality, aging, correlation, Human

## Abstract

A comparison of nine different approaches over a period of 20 years reveals the most promising indicators for biological age.

**Related research article** Li X, Ploner A, Wang Y, Magnusson PKE, Reynolds C, Finkel D, Pedersen NL, Jylhävä J, Hägg S. 2020. Longitudinal trajectories, correlations and mortality associations of nine biological ages across 20-years follow-up. *eLife* **9**:e51507. doi: 10.7554/eLife.51507

To see if treatments to ward off aging work, first we need a way to measure biological age reliably (Moskalev, 2019). Biological age is a complex parameter involving the calendar age of a person, their health as relating to their age, and medical signs of when they might die of old age. Historically, the first estimates of biological age were based on markers that could be measured in the clinic (such as inflammation, glucose resistance, and endocrine markers) and on functional tests (such as cognitive function and cardiorespiratory fitness; reviewed in Jia et al., 2017). Such markers have a direct clinical interpretation, but even if they predict mortality better than passport age, it is unclear to what extent they measure biological aging itself, rather than health deterioration for other reasons. Additionally, these markers often only work well as averaged indicators in very large samples, and vary a lot between individuals. However, it may be possible to overcome these limitations by using artificial intelligence to generate models using several aging biomarkers (Zhavoronkov et al., 2019).

Other approaches, based on a deeper understanding of the molecular and cellular causes of aging, include measuring the levels of p16 (a marker for cellular senescence or when a cell stops dividing) and measuring the telomere length in leukocytes (biological age increases as telomere length decreases; Waaijer et al., 2012; Epel et al., 2009). Theoretically, these markers should be more sensitive to early signs of aging (as opposed to mortality and frailty) but, similar to clinical markers for individual patients, they lack robustness and reproducibility. This is because aging is a multi-level process, so markers of individual mechanisms cannot cover all its aspects.

A third approach is to use ‘omics’ (that is, to analyze the transcriptome, methylome, proteome and metabolome). Changes in the ‘omes’ are the result of changes in the organism at different levels, making them a useful way to approach the complexity of the aging process. Using this approach, there is no single biological age, but rather a metabolic, proteomic or methylome age. Multi-omics approaches have also been used to assess the rate of aging (Solovev et al., 2020).

Within omics, analyses of DNA methylation or epigenetic clocks are the most robust indicator of age-related changes and have become a booming area of research (Bell et al., 2019). But questions still remain. To what extent are epigenetic clocks a function of age, and to what extent part of biological aging? How does the epigenome change with age? How closely are epigenetic clocks associated with mortality? Is it possible to reverse the epigenetic age, for example through lifestyle changes or interventions? Diet, exercise, education and lifestyle factors seem to be able to influence the rate of aging according to the epigenetic clock (Quach et al., 2017; Gensous et al., 2019; Sae-Lee et al., 2018). Certain drugs can slow down the epigenetic clock in cells cultured in the lab (Horvath et al., 2019) and certain treatments have also proved to be effective in vivo (Chen et al., 2019; Fahy et al., 2019).

Now, in eLife, Sara Hägg from the Karolinska Institute and colleagues from the University of California Riverside, Indiana University Southeast and Jönköping University – with Xia Li as first author – study how nine different methods to estimate biological age change over time in a cohort of 845 middle-aged and older individuals from Sweden who were studied over a period of 20 years (Li et al., 2020). Three of the biological ages measured were functional (cognitive function, functional aging index, and frailty index) and four were based on the levels of DNA methylation (called Horvath, Hannum, PhenoAge and GrimAge). The other two were telomere length (measured by qPCR) and physiological age (calculated as a composite score of clinical measurements such as body-mass index or waist circumference, and blood biomarkers such as hemoglobin or cholesterol).

This study is unique because it compares several approaches at once and evaluates how the measurements change over time: functional data and biological samples were collected nine times between 1986 and 2014. The profiles for the three functional measurements indicated that accelerated aging started around the age of 70, whereas the other biological ages showed linear growth with time.

The authors found sex differences in the mean levels of the different biological ages. Women exhibited longer telomere length and lower DNA methylation age compared to men, but also averaged higher in two of the three functional estimates. Telomere length showed the weakest correlations with both chronological age and with the other measurements. The highest correlations were between two of the DNA methylation ages (Horvath and Hannum), and between the functional aging index and the other two functional biological ages. Regarding the ability of biological ages to predict age-related mortality, one of the functional estimates (frailty index) and one of the methylation clocks (GrimAge) were the best predictors, while telomere length was the worst.

These results indicate that methylation age and frailty index are the most promising approaches to estimating biological age, and underline the value of assessing these estimates overtime in the same population.
